# Long bone fracture reduction and deformity correction using the hexapod external fixator with a new method: a feasible study and preliminary results

**DOI:** 10.1186/s12891-021-04097-9

**Published:** 2021-02-24

**Authors:** Yanshi Liu, Hong Li, Jialin Liu, Xingpeng Zhang, Maimaiaili Yushan, Zhenhui Liu, Chuang Ma, Aihemaitijiang Yusufu

**Affiliations:** 1 Department of Microrepair and Reconstruction, The First Affiliated Hospital of Xinjiang Medical University, Urumqi, Xinjiang China; 2 Department of Orthopedics, Zigong Fourth People’s Hospital, Zigong, Sichuan China; 3 Department of Prosthodontics, The First Affiliated Hospital of Xinjiang Medical University, Urumqi, Xinjiang China; 4 Department of Orthopedics, Shanghai Pudong New Area People’s Hospital, Shanghai, China

**Keywords:** Computer‐assisted, Fracture reduction, Hexapod external fixation, Taylor spatial frame, Three‐dimensional reconstruction

## Abstract

**Background:**

The hexapod external fixator (HEF), such as the Taylor spatial frame (TSF), offering the ability of multidirectional deformities correction without changing the structure, whereas there are so many parameters for surgeons to measure and subjective errors will occur inevitably. The purpose of this study was to evaluate the effectiveness of a new method based on computer-assisted three-dimensional (3D) reconstruction and hexapod external fixator for long bone fracture reduction and deformity correction without calculating the parameters needed by the traditional usage.

**Methods:**

This retrospective study consists of 25 patients with high-energy tibial diaphyseal fractures treated by the HEF at our institution from January 2016 to June 2018, including 22 males and 3 females with a mean age of 42 years (range 14–63 years). Hexapod external fixator treatments were conducted to manage the multiplanar posttraumatic deformities with/without poor soft-tissue that were not suitable for internal fixation. Computer-assisted 3D reconstruction and trajectory planning of the reduction by Mimics were applied to perform virtual fracture reduction and deformity correction. The electronic prescription derived from the length changes of the six struts were calculated by SolidWorks. Fracture reduction was conducted by adjusting the lengths of the six struts according to the electronic prescription. Effectiveness was evaluated by the standard anteroposterior (AP) and lateral X-rays after reduction.

**Results:**

All patients acquired excellent functional reduction and achieved bone union in our study. After correction, the mean translation (1.0 ± 1.1 mm) and angulation (0.8 ± 1.2°) on the coronal plane, mean translation (0.8 ± 1.0 mm) and angulation (0.3 ± 0.8°) on the sagittal plane were all less than those (6.1 ± 4.9 mm, 5.2 ± 3.2°, 4.2 ± 3.5 mm, 4.0 ± 2.5°) before correction (*P* < 0.05).

**Conclusions:**

The computer-assisted three-dimensional reconstruction and hexapod external fixator-based method allows surgeons to conduct long bone fracture reduction and deformity correction without calculating the parameters needed by the traditional usage. This method is suggested to apply in those unusually complex cases with extensive soft tissue damage and where internal fixation is impossible or inadvisable.

## Background

External fixation played a crucial role in treating open fractures, bone infections, limb deformities, or bone defects, especially those with extensive soft tissue damage [1–4]. Prior to external fixation, it was crucial to realign the fracture fragments. The anatomic reduction included advantages of less time to union [5], lower possibility of malunion and nonunion [6], and satisfactory function and appearance of the limb [7]. Given that the three-dimensional(3D) spatial deformities in actuality, the conventional approach for fracture reduction using an external fixator based on the two-dimensional(2D) fluoroscopic with a limited field of view and static remains a challenge, often results in the surgeons making a compromise between the quality of reduction and time taken [8, 9].

The hexapod external fixator (HEF), such as the Taylor spatial frame (TSF), combining gradual distraction principles with deformity analysis conducted by a software [3, 10]. This frame is an symmetric configuration of the Stewart platform, consisting of 2 rings or partial rings connected by 6 telescopic struts at special universal joints [11]. It offers the advantages of multiplanar deformities correction without converting the frame [3, 10, 12–14], and makes the reduction process more objective and experience-independent. However, the effectiveness depends on the measurement accuracy of the input parameters which calculated by 2D X-rays. In general usage, for TSF, surgeons must measure 13 parameters precisely, including six deformity parameters, four mounting parameters, and three frame parameters. There are many factors that can affect the effectiveness, and errors in any one parameter will affect the entire preoperative plan. Of all these parameters, the axial rotational deformity is easier to mismeasurement due to the 2D radiographs can’t contain axial spatial information, and it is traditionally determined by clinical examination [15–17]. Additionally, the measured anatomical and mechanical angles may be influenced by limb rotation on radiographs significantly [18, 19].

These practical constraints ground the premise of the present study. Based on previously published data and our previous exploration, the purpose of this study was to propose a new method based on computer-assisted 3D reconstruction and hexapod external fixator for long bone fracture reduction and deformity correction without calculating the parameters needed by the traditional usage.

## Methods

This retrospective study consisted of 25 trauma patients with tibial diaphyseal fracture treated by the hexapod external fixator at our institution from January 2016 to June 2018, including 22 males and 3 females with an average age of 42 years (range 14–63 years). Informed consent was acquired from all patients for their information to be documented and published in this study. The Ethical Committee of our institution approved this present study.

Hexapod external fixator treatments were conducted to manage the multiplanar posttraumatic deformities with/without poor soft-tissue that were not suitable for internal fixation. Patients with open fractures or polytrauma treated by the hexapod external fixator and who suffered postoperative deformities with an angular deformity greater than 5° in any anatomical plane or a translational deformity greater than 10mm were included[9]. Fractures associated with vascular and nerve injury, pathological fractures, age > 65 years, diseases that are harmful to bone healing (such as diabetes, osteoporosis, etc.), and patients with poor compliance were excluded.

### Planning procedure of electronic prescription

Surgical procedures were conducted by the same surgical team. The anteroposterior (AP) and lateral radiographs after operation were taken to assess the postoperative deformities. When there were significant deformities that accord with the aforementioned inclusion criteria, the postoperative reduction was performed.

The postoperative computed tomographic (CT) data of the bilateral extremities were imported into Mimics 17.0 (Materialise, Belgium) for 3D reconstruction, registration, and virtual trajectory planning of the reduction. The reference (proximal) ring and distal ring which contains the spatial positional information at each critical point in the trajectory were exported as STL (Standard Template Library) files by Mimics and then imported into SolidWorks 2016 (Dassault Systemes, USA) to calculate the lengths of the six struts.

The electronic prescription was derived from the length changes of the six struts.

### Technique and case example

**Fig. 1:**
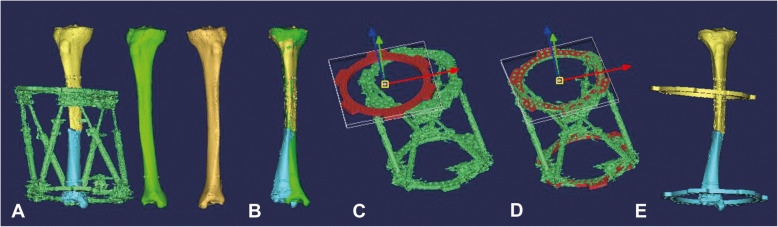
3D reconstruction and registration of the whole model. **a** 3D reconstruction of the bilateral tibia and the TSF. The green tibia is the mirror of the contralateral tibia. **b** Registration of the proximal bony fragment and mirrored image of the contralateral tibia. **c, d** Registration of the standard ring and the 3D model of the actual ring. **e** Merge of the bony fragment and the corresponding ring

**Fig. 2:**
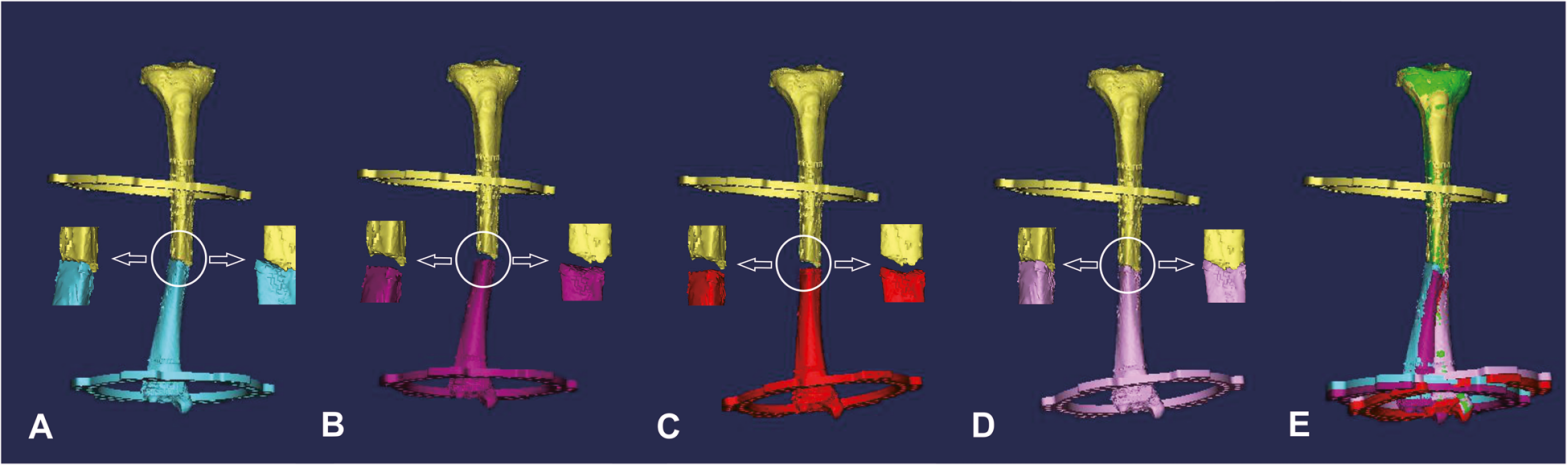
Flowchart of the trajectory planning for fracture reduction. **a** Original position of the whole model. **b** Extension of the distal part. **c** Rotation of the distal part. **d** Reduction of the distal part. **e** Schematic image of the whole planning

**Fig. 3:**
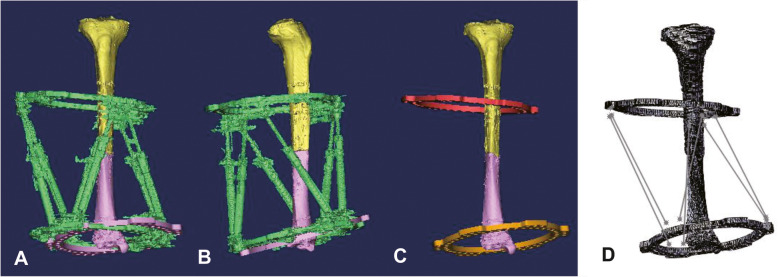
Position changes of the distal ring before and after reduction. **a** AP view of the whole model. **b** Lateral view of the whole model. **c** Separation of the bony fragment and the corresponding ring from the two parts. **d** Calculate the length of the six struts in SolidWorks

**Fig. 4:**
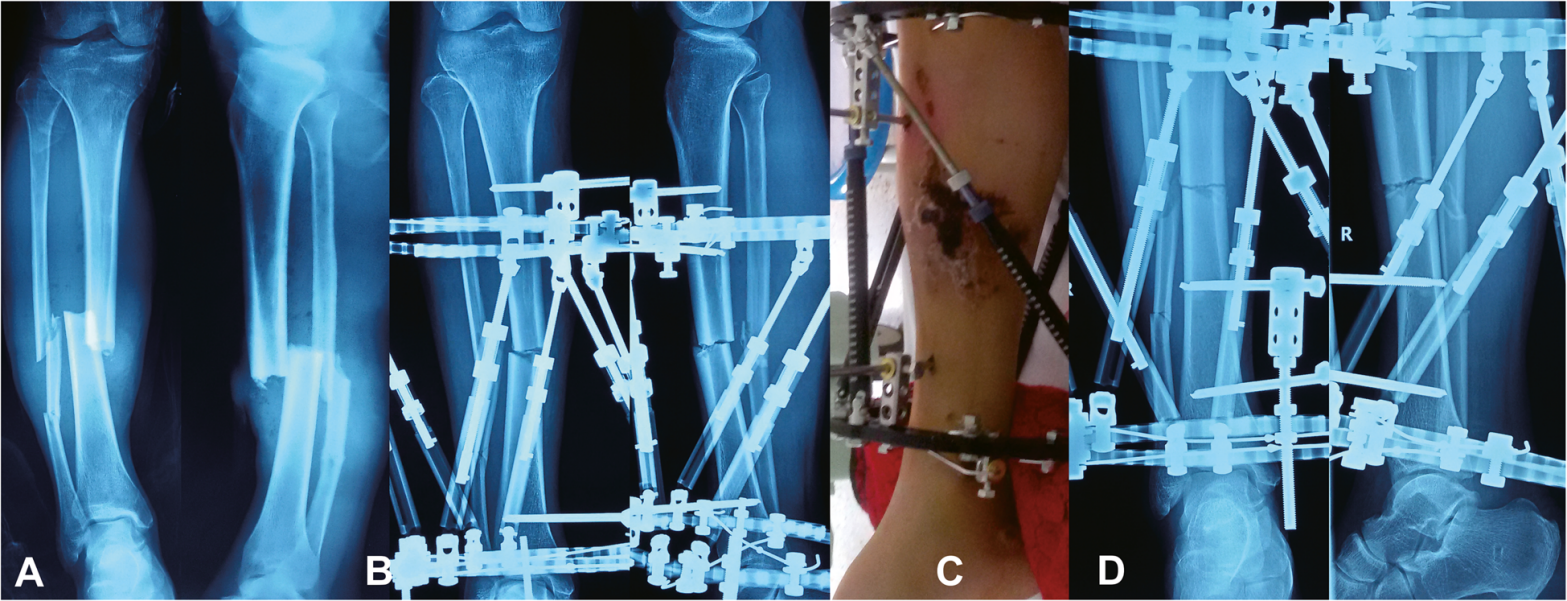
Images of a 54-year-old man with posttraumatic multidimensional deformities in tibia and fibula treated by the hexapod external fixation (HEF). **a** Posttraumatic AP and lateral views of X-rays. **b** Radiographs immediately after application of HEF. **c** Patient in HEF with an open wound and preoperative hemorrhagic fracture blisters seen. **d** Radiographs after final correction

A 54-year-old man suffered open tibiofibular fractures in a traffic accident with multiplanar posttraumatic deformities. He had severe hemorrhagic fracture blisters on presentation to our institute 2 days after the injury. In this case, computer-assisted 3D reconstruction and virtual trajectory planning of the reduction with Mimics were applied to perform fracture reduction and deformity correction based on hexapod external fixation (Figs. 1, 2, 3 and 4).

The 3D model of the injured limb, contralateral healthy limb, and external fixator were reconstructed based on postoperative CT data by threshold division, bone separation, and image editing in Mimics. The proximal bony fragment and ring were defined as reference bony fragment and reference ring, which were motionless relatively. The mirrored 3D model of the contralateral healthy limb was regarded as the reference for reduction, and it was registered with the reference bony fragment. To eliminate the visual interference caused by metal artifacts on the actual external frame, two standard ring models which are the same size as the actual ring of the HEF were created in STL format by SolidWorks, loading them into Mimics and registered with the 3D model of the actual proximal and distal ring. The bony fragment and its corresponding standard ring were merged into one part, the whole model was divided into two parts, and the position of the bony fragment relative to its corresponding standard ring was locked in each part (Fig. 1).

The proximal part was motionless relatively. The virtual trajectory of fracture reduction was planned by adjusting the spatial position of the distal part with the function of move and rotation in Mimics. Three steps with three critical points performed the reduction. Firstly, the distal part was moved at an appropriate distance to the distal end, providing sufficient space for the relative movement of the two bony ends. Secondly, the distal part was moved and rotated in multiple planes to correct the spatial deformities referred to the mirrored 3D model of the contralateral healthy limb. Thirdly, the bony end of the two parts was docked with a comprehensive reference to the profile of the docking site and the mirrored 3D model of the contralateral healthy limb (Fig. 2).

The relative position of the bony fragment and its corresponding ring was locked in each part, the position changes in the bony fragment could be replaced by those in ring. The relative position information of the two bony fragments at each critical point during the procedure of virtual reduction was obtained. Each part was separated into bony fragment and ring at every critical point, and these rings were named in order and saved. The two rings of the same group which contain the information of spatial position were imported into SolidWorks for an assembly model, and six virtual struts were created based on the actual situation in this assembly model. The lengths of the six virtual struts were the electronic prescription needed by HEF (Fig. 3).

### Reduction management and effectiveness evaluation

Fracture reduction was conducted by adjusting the lengths of the six struts according to the electronic prescription. The effectiveness was evaluated by the translation and angulation in the coronal and sagittal plane, according to the standard AP and lateral X-rays after reduction.

The patients were followed up weekly until total corrections achieved, and then monthly during the mineralization time. Complications that occurred intraoperatively and during postoperative treatment were classified according to Paley [20]. The HEF was removed when radiographs showed sufficient union (corticalization in 3 of 4 cortices) and a clinical assessment was made. All patients were closely followed up at a minimum of 12 months after removing the HEF.

### Statistical analysis

The SPSS 22.0 (IBM Corp, USA) software was used to perform statistical analysis. Continuous variables were analyzed by paired-samples T-tests and expressed as the mean and standard deviation (SD). The Statistical significance was set at *P* < 0.05.

## Results

**Fig. 5:**
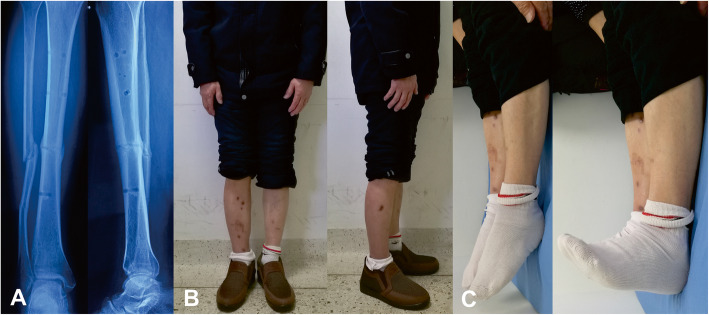
Follow-up images of the same patient after removing the HEF. **a** Radiographs one month later. **b, c** Clinical images of the patient, obtained at 13 months after HEF removal

Table 1 shows the differences before and after reduction. All patients achieved an excellent functional reduction in our study. The mean translation and angulation on the coronal plane before correction were 6.1 ± 4.9 mm and 5.2 ± 3.2°. The mean translation and angulation on the sagittal plane before correction were 4.2 ± 3.5 mm and 4.0 ± 2.5°. After correction, the mean translation and angulation were 1.0 ± 1.1 mm and 0.8 ± 1.2°on the coronal plane, while that were 0.8 ± 1.0 mm and 0.3 ± 0.8°on the sagittal plane. There were statistically significant differences before and after correction (*p* < 0.05). (Typical cases were shown in Figs. 4 and 5)

**Table 1:** Comparison of the residual deformities before and after correction

Parameters	Before	After	t	*P*-value
Coronal plane translation	6.1 ± 4.9	1.0 ± 1.1	5.614	*P* < 0.001
Coronal plane angulation	5.2 ± 3.2	0.8 ± 1.2	7.426	*P* < 0.001
Sagittal plane translation	4.2 ± 3.5	0.8 ± 1.0	5.006	*P* < 0.001
Sagittal plane angulation	4.0 ± 2.5	0.3 ± 0.8	6.942	*P* < 0.001

Values are presented as mean ± SD. Translation in mm and angulation in degree (°).

All patients (100 %) acquired complete bone union with a mean time of 26 weeks (range 16 to 40 weeks) and were successfully followed for a mean of 15 weeks (range 12 to 25 weeks) after removing the HEF. There were no intraoperative complications. Twelve patients suffered superficial pin tract infection and successfully solved by daily pin site care and oral antibiotics. Delayed union was observed in one case and finally treated by the “accordian” technique; the TSF was thereby applied for 40 weeks. One patient suffered ankle joint stiffness after removing the HEF, and successfully managed by a surgical release. There was no loss of reduction, no neurovascular injury, and no refracture. All the patients were able to perform activities of daily life without significant difficulty when last seen.

## Discussion

The poor reduction is a significant risk factor for fracture healing. Fractures that healed with an angular deformity greater than 5°in any anatomical plane or a translational deformity greater than 10mm are regarded as malunion [9]. Even minor alteration of the mechanical axis has been demonstrated to promote the progression to arthritis in both knee and ankle significantly [21, 22]. As generally accepted, the anatomic reduction is vital for fracture treatments.

To improve reduction accuracy, many computer-assisted techniques have been developed. Koo et al. [9, 23] developed an algorithm to perform closed fracture reduction using a unilateral external fixator, whereas there lacked a device that could facilitate its implementation in clinical practice. Hofstetter et al. [24, 25] and Weil et al. [26] developed fluoroscopy-based navigation techniques in which the bone fragments are displayed through superimposed line graphics to provide the real-time spatial relationship between the bone fragments. Navigation systems based on CT were also developed for accurate fracture reduction [27, 28].

For trauma-control and definitive management of polytrauma with complex fractures, the external fixation played a crucial role in recent years [2, 29]. Alhammoud et al. [2] solely applied different uniplanar or multiplanar external fixators as primary and definitive management for a large number of 955 patients, concluding that external fixation was a reliable method for open long bone shaft fractures caused by high energy trauma. Testa et al. [29] conducted a monoaxial external fixation treatment in 83 patients with femoral shaft fractures. The satisfactory outcomes manifested it was an ideal method, including advantages of minimal additional operative injury and an acceptable complication rate.

Multidirectional deformities can be corrected simultaneously by the hexapod external fixation without changing the frame [3, 4, 11–13, 16, 30, 31]. However, the effectiveness can be affected by some inherent limitations. The postoperative adjustments are based on the measurements of standard orthogonal AP and lateral radiographic [32], whereas they are often conducted subjectively by radiologists. Besides, position the limbs for taking the orthogonal radiographs is challenging, especially in patients with severe deformities. Lots of published data had demonstrated that the mechanical axis, the mechanical femorotibial axis (mFTA), the femoral anatomic mechanical angle (AMA), the mechanical lateral distal femoral angle (mLDFA), and the mechanical medial proximal tibial angle (mMPTA) would be influenced by the radiographs performed with malrotation of the limb [18, 19, 33, 34]. It is generally accepted that malrotation on radiographs can lead to wrong anatomical measurements, however it is difficult to assess this malrotation. As above, the degree of deformity correction and the treatment success will be largely influenced by the rotation-related changes in the measured anatomic alignment.

Moreover, the reference ring must be perpendicular to the reference bony fragment in AP and lateral view, which is also a challenge for surgeons during the surgical installation. The whole reference ring must be visible on the radiographs when measuring the mounting parameters, while the lateral border of the reference ring was incomplete or obscured in most radiographs in our retrospective analysis. The spatial information, especially for the rotational deformities, can’t be obtained from the radiographs which are shown in the 2D planes, and they were calculated by clinical examination in traditional usage which is not accurate [17]. Additionally, there are 13 parameters for surgeons to measure and subjective errors will occur inevitably. The majority of residual deformities have been reported due to inaccurate mounting parameters [35]. The aforementioned situations may result in a time-consuming process, which is often followed by repeated radiographs including further radiation exposure for the patient.

To prevent the miscalculation of the parameters needed by the hexapod external fixation system, several techniques have been proposed [32, 35–37]. Kucukkaya et al. [35] calculated the mounting parameters using the tomographic images in CT and demonstrated its advantages, especially in deformities with a rotational deformity. Gantsoudes et al. [37] declared that intraoperative measurement is easily reproducible in the operating room and allows for accurate measurement of the mounting parameters. Ahrend et al. [38] enhanced the standard radiographic procedure using a rotation rod to control limb rotation before taking radiographs, and their results showed lower variability of rotation, more reproducibility, and better comparability on radiographs. However, the aforementioned techniques are all dependent on the measurements in 2D planes.

The presented new method used postoperative CT scans to establish the relationship between the bone and ring, and then replaced the spatial changes in bony fragments with that in the two rings. In our study, there was no need to conduct orthogonal AP and lateral X-rays to measure so many parameters for surgeons, and this way may be a potential benefit to prevent wrong measurements. Besides, surgeons conducted virtual reduction under the direct vision by Mimics, and the three-point trajectory of “extend-rotate-reduct” can easily avoid the collision and interference between the irregular bony end in the process of fracture reduction. In this method, the most important one for surgeons is just keeping the stable fixation in the bony fragment and its corresponding ring, and there was no need to ensure that the reference ring was perpendicular to the reference bony fragment. According to our experience, this method also can be extended for fracture reduction and deformity correction in other long bones.

The present study had several limitations. Firstly, the new method here may be limited by the two software, Mimics and SolidWorks, which required the surgeons to have a basic knowledge of these software. The procedures are tedious and time-consuming in inexperienced hands, especially under the influence of metal artifacts for 3D reconstruction. A practical software that contains the aforementioned comprehensive function needs to be developed for these limitations. Secondly, the control group in which conduct parameters measurement on 2D radiographs is needed to compare the accuracy of the present method, whereas the clinical outcomes have demonstrated the effectiveness of our new method. Thirdly, considering the higher radiologic exposure in CT than X-rays, our method is suggested to apply in severe open fractures or those unusually complex cases with severe deformities, especially in those with extensive soft tissue damage and internal fixation is impossible or inadvisable.

## Conclusions

The computer-assisted three-dimensional reconstruction and hexapod external fixator-based method allows surgeons to conduct long bone fracture reduction and deformity correction without calculating the parameters needed by the traditional usage. This method is suggested to apply in those unusually complex cases with severe deformities, especially in those with extensive soft tissue damage and where internal fixation is impossible or inadvisable.

## Data Availability

The datasets analyzed during the current study are available from the corresponding author on reasonable request.

## Abbreviations

HEF: hexapod external fixator
TSF: Taylor spatial frame
3D: three-dimensional
2D: two-dimensional
AP: anteroposterior
CT: computed tomographic
STL: Standard Template Library
SD: standard deviation
